# Formulation and *In-vitro* Evaluation of Orally Disintegrating Tablets of Olanzapine-2-Hydroxypropyl-β-Cyclodextrin Inclusion Complex

**Published:** 2010

**Authors:** Kulkarni Ajit Shankarrao, Ghadge Dhairysheel Mahadeo, Kokate Pankaj Balavantrao

**Affiliations:** *Depatment of Pharmaceutics, Satara College of Pharmacy, Satara, M.S. India.*

**Keywords:** Orally disintegrating tablets, Olanzapine, Olanzapine-2-hydroxypropyl-β- cyclodextrin inclusion complex, Phase solubility analysis

## Abstract

The aim of this study was to design orally disintegrating tablets of Olanzapine and to complex Olanzapine with 2-hydroxypropyl-β- cyclodextrin with special emphasis on disintegration and dissolution studies. Phase solubility studies demonstrated the formation of 1:1 molar inclusion complex by kneading method. Tablets were prepared by using superdisintegrants namely, sodium starch glycolate, croscarmellose sodium, crospovidone, tulsion 339, and indion 414. Complex was characterized using infrared spectroscopy, drug content estimation, saturated solubility study, diffrerential scanning calorimetry and X-ray diffractometry. 5% w/w croscarmellose sodium showed the minimum disintegration time 39 ± 1.76 sec and *in-vitro *drug release 99.19 ± 0.18% within 6 min. In general, solubility of Olanzapine can be improved by complexing with 2-hydroxypropyl-β- cyclodextrin. Croscarmellose sodium can be used for faster disintegration of tablets.

## Introduction

Oral drug delivery remains the preferred route of drug delivery (1). Recent developments in technology have presented viable dosage alternatives for patients who may have difficulty in swallowing tablets or liquids. Traditional tablets and capsules administered with 8–OZ glass of water may be inconvenient or impractical for some patients. For example, a very elderly patient may not be able to swallow a daily dose of antidepressant, an eight-year-old with allergies could use a more convenient dosage form than an antihistamine syrup, a schizophrenic patient can hide a conventional tablet under his or her tongue to avoid its daily dose of an atypical anti-psychotic (2). Orally disintegrating tablets (ODTs) are a perfect fit for all these patients. 

Some tablets are designed to dissolve in saliva remarkably fast within a few seconds and are the true ODTs. Other tablets contain agents to enhance the rate of tablet disintegration in oral cavity and are more appropriately termed as ODTs (3). 

Olanzapine is classified as a thiobenzodiazepines. It is an atypical antipsychotic drug used in the treatment of schizophrenia. It is practically insoluble in water, having only 60% oral bioavailability. Olanzapine undergoes extensive first pass metabolism (4). Some schizophrenic patients hide a conventional tablet under their tongue to avoid its daily dose of an atypical antipsychotic. Also schizophrenic patients with dysphagia are not able to swallow conventional olanzapine tablet. To overcome this problem an attempt was made to formulate and evaluate ODTs of olanzapine-2-hydroxypropyl-β-cyclodextrin inclusion complex. Inclusion complex of olanzapine with 2-hydroxypropyl-β-cyclodextrin was made to improve the aqueous solubility of olanzapine and to enhance dissolution rate of olanzapine. It may enhance the pregastric absorption of olanzapine. 2-hydroxypropyl-β-cyclodextrin may act as channel forming agent because it helps in quick disintegration of tablets and may act as permeation enhancer to pass olanzapine through oral mucosa (5-8). Tablets were prepared by using 2-hydroxypropyl-β-cyclodextrin and five superdisintegrants, namely as SSG, croscarmellose sodium, crospovidone, tulsion 339 and indion 414. Superdisintegrants are added to facilitate drug release and consequently improve the solubility of olanzapine (9,10). Tablets were prepared by using direct compression technique. The simplicity and cost effectiveness of the direct compression technique have positioned direct compression as an alternative to granulation technologies. To the best of our knowledge, no work has been reported on olanzapine complex with 2-hydroxypropyl-β-cyclodextrin.

## Experimental

*Materials*

Olanzapine was obtained as a gift sample form Okasa Pharma Pvt. Ltd., (Satara, India) and from Cipla Ltd. (Kurkumbh, India), 2-hydroxypropyl-β-cyclodextrin was generously donated by Signet Chemical corporation, (Mumbai, India), Aspartame was obtained as a gift sample from Sun and Kingly Pharma Pvt. Ltd. (Satara, India), Tulsion 339 was a gift from Thermax India Ltd. (Pune, India), Indion 414 was kindly donated by Ion Exchange India Ltd. (Mumbai, India) and All other excipients ethanol, avicel PH102, mannitol, SSG, croscarmellose sodium, crospovidone, vanilin dry flavor, aerosil and Mg stearate were procured from Rajesh Chemicals (Mumbai, India). All reagents and solvents were of analytical grade.

*Methods*

*Phase solubility analysis for olanzapine*

Phase solubility studies were performed to determine stoichiometric proportions. This data was used to determine stability constant of complex. For this purpose, the stock solution of 12 mM of 2-hydroxypropyl-β-cyclodextrin was prepared using distilled water. This stock solution was diluted with distilled water to give molar solutions in the range of 0 mM to 12 mM of 2-hydroxypropyl-β-cyclodextrin. 5 mL of each molar solution was filled in a screw capped vials and the excess quantity of olanzapine was added to each vial separately. The vials were kept for shaking at ambient temperature for 24 h using a lab shaker (Remi). After the complete equilibration, the supernatant solutions were collected carefully and filtered using Whatman filter paper (No. 41). The concentration of olanzapine in filtered solutions was determined using UV visible spectrophotometer (UV-1700 Schimadzu spectrophotometer, Tokyo, Japan) at 253.5 nm. No change in λ max of drug was observed after complexing with cyclodextrins. The graph was plotted against drug concentration vs. concentration of 2-hydroxypropyl-β-cyclodextrin.

The blanks were prepared using the same concentration of 2-hydroxypropyl-β-cyclodextrin in distilled water so as to cancel out any absorbances that may be exhibited by 2-hydroxypropyl-β-cyclodextrin molecules (11, 12).

Concentration = 1/Slope × Absorbance

The stability constant of the olanzapine 2-hydroxypropyl-β-cyclodextrin inclusion complex was determined from the slope of linear portion of the curves and intrinsic solubility of olanzapine in an aqueous solution using equation:

Stability constant = Slope/Intercept (1-Slope)

*Preparation of inclusion complex*

*Kneading method*

Required quantities of olanzapine and cyclodextrin was weighed accurately in 1:1 molar ratio. A homogenous paste of cyclodextrin and olanzapine was prepared in mortar by adding ethanol in small quantities. An appropriate quantity of ethanol was added to maintain suitable consistency of paste. The paste was kneaded for 1 h and then dried in hot air oven (Singhal scientific) at 45-50°C for 3 h. The dried complex was powdered and passed through sieve No. 60 and stored in airtight containers till further use (13, 14).

*Characterization of Olanzapine-2-hydroxypropyl- β-cyclodextrin inclusion complex*

Inclusion complex was characterized and evaluated using following techniques. 

*Drug content estimation*

Olanzapine-2-hydroxypropyl-β-cyclodextrin complex, equivalent to 10 mg of drug, was accurately weighed and added in to 100 mL volumetric flask. To this, 100 mL ethanol was added.

This solution was stirred for 60 min, till the entire drug leached out. The solution was filtered and 1 mL was withdrawn from this solution and added in to 10 mL volumetric flask and volume was made to 10 mL (10 μg/mL) with phosphate buffer pH 6.8. Drug content was estimated UV spectrophotometrically at 253.5 nm, using phosphate buffer pH 6.8 as blank. 

*Saturation solubility studies*

Saturation solubility study was performed according to method reported by Higuchi and Connors (11). Excess quantities of inclusion complex were added to 25 mL distilled water in a stoppered conical flasks and mixtures were shaken for 24 h in rotary flask shaker.

After shaking, to achieve equilibrium, 2 mL aliquots were withdrawn at 1 h intervals and filtered through Whatman filter paper No. 41. The filtrate was analyzed spectrophotometrically at 253.5 nm. Shaking was continued until three consecutive readings were the same (13). 

*IR spectral analysis*

Infrared spectra of pure olanzapine, 2-hydroxypropyl-β-cyclodextrin and inclusion compelx were recorded by KBr method using fourier transform infrared spectrophotometer (spectrum one, by Perkin-Elmer, USA). A baseline correction was made by using dried potassium bromide and then spectras of dried mixtures of olanzapine, 2-hydroxypropyl-β-cyclodextrin and inclusion complex with potassium bromide were recorded. Scanning was done from 450 -4000 cm^-1^.

*X-ray diffractometery (XRD)*

X-ray diffraction patterns of pure olanzapine, 2-hydroxypropyl-β-cyclodextrin and inclusion complex were recorded using (Philips-PW3710, Holland) X-ray diffractometer with a copper target, voltage 40 Kv, current 30 MA at a scanning speed of 0.30 °C /min. 

*Differential scanning calorimetry (DSC)*

Thermograms of pure Olanzapine, 2-hydroxypropyl-β- cyclodextrin and inclusion complex were recorded. (TA instruments Inc, SDT 2960, USA). About 5 mg of samples were sealed in aluminum pans and heated at a rate of 20 °C/min. from 20-250 °C under nitrogen atmosphere of 100 mL/min flow rate (15).

*Dissolution study of olanzapine and its inclusion complex*

*In-vitro *dissolution of olanzapine (10 mg) and its inclusion complex equivalent to 10 mg of olanzapine was studied using Veego scientific, USP tablet dissolution apparatus type II. The dissolution was carried out in 900 mL phosphate buffer pH 6.8. at 37 ± 0.5 °C, at 50 rpm. 10 mL aliquots were withdrawn at specific time interval and filtered using Whatman filter paper No. 41. Absorbance of the filtered solution was checked UV spectrophotometrically at 253.5 nm and the drug content was determined. Sink conditions were maintained throughout the study. All studies were carried out in triplicate (3). 

*Formulation and evaluation of tablets*

*Formulation of tablets*

ODTs of olanzapine were prepared by direct compression method according to the formula given in Table 1. Various superdisinterants with different concentrations were used such as SSG, croscarmellose sodium, crospovidone, tulsion 339 and indion 414. olanzapine-2-hydroxypropyl-β-cyclodextrin inclusion complex and Avicel PH102 were passed through sieve No. 60 and mixed homogeneously for 15 min (blend I). Mannitol, superdisintegrant, aspartame, vanillin and aerosil were passed through sieve No. 40 and mixed homogeneously for 10 min (called as blend II). Blend II was mixed with blend I and mixing was continued for 1 h.

**Table 1:** Formulation design equivalent to 10 mg of olanzapine

**Name of ingredients Qty.in mg.**	**Control**	**A1**	**A2**	**A3**	**B1**	**B2**	**B3**	**C1**	**C2**	**C3**	**D1**	**D2**	**D3**	**E1**	**E2**	**E3**
Inclusion complex	54.80	54.80	54.80	54.80	54.80	54.80	54.80	54.80	54.80	54.80	54.80	54.80	54.80	54.80	54.80	54.80
Superdisintegrant	-	14	15.75	17.5	5.25	7	8.75	-	-	-	5.25	7	8.75	5.25	7	8.75
Avicel PH102	75.45	61.45	59.7	57.95	70.2	68.45	66.7	70.2	68.45	66.7	70.2	68.45	66.7	70.2	68.45	66.7
Aspartame	1	1	1	1	1	1	1	1	1	1	1	1	1	1	1	1
Mannitol	41	41	41	41	41	41	41	41	41	41	41	41	41	41	41	41
Vanilin dry flavor	1	1	1	1	1	1	1	1	1	1	1	1	1	1		1
Aerosil	0.875	0.875	0.875	0.875	0.875	0.875	0.875	0.875	0.875	0.875	0.875	0.875	0.875	0.875	0.875	0.875
Mg.stearate	0.875	0.875	0.875	0.875	0.875	0.875	0.875	0.875	0.875	0.875	0.875	0.875	0.875	0.875	0.875	0.875
Tablet weight	175	175	175	175	175	175	175	175	175	175	175	175	175	175	175	175

Finally, Magnesium stearate was added and mixed for 1 min. This blend was compressed using 8 mm size flat faced punch on (Cadmach) single punch compression machine. 

Control formulation was formulated without adding superdisintegrant. Mannitol improves patient compliance by imparting cool sensation and sweet mild taste. It flows well and improves flow properties of other materials (16). Aspartame was used as sweetener. It enhanced flavor systems and also used as taste masking agent. The approximate sweetening power is 180-200 times to that sucrose. Stability of aspartame can be enhanced by addition of cyclodextrin, vanilin dry flavor was used as a flavoring agent (17). It imparts a characteristic taste and odor of natural vanilla. Aerosil was used as glidant, magnesium stearate was used as lubricant (17). 

*Evaluation of tablets*

*Diameter*

It was measured by digital vernier caliper. It is expressed in mm. 

*Thickness*

It was determined by using digital vernier caliper. It is expressed in mm.

*Crushing strength*

Tablet crushing load, which is the force required to break a tablet by compression in radial (diametrical) direction was measured by using tablet hardness tester. Crushing strength for crushing (T) was calculated using the following formula.

T = 2F/ΠDT F = Crushing load 

D = Diameter 

T = Thickness

*Hardness*

The hardness of the tablets was determined using Monsanto hardness tester. It is expressed in kg/cm^2^ (18). 

*Friability*

Friability of the tablets was determined using Roche friabilator. It is expressed in % (18). 

*Disintegration time*

It was measured by two methods 

I) Using USP disintegration test apparatus.

II) Using modified disintegration test apparatus.

**Figure 1:**
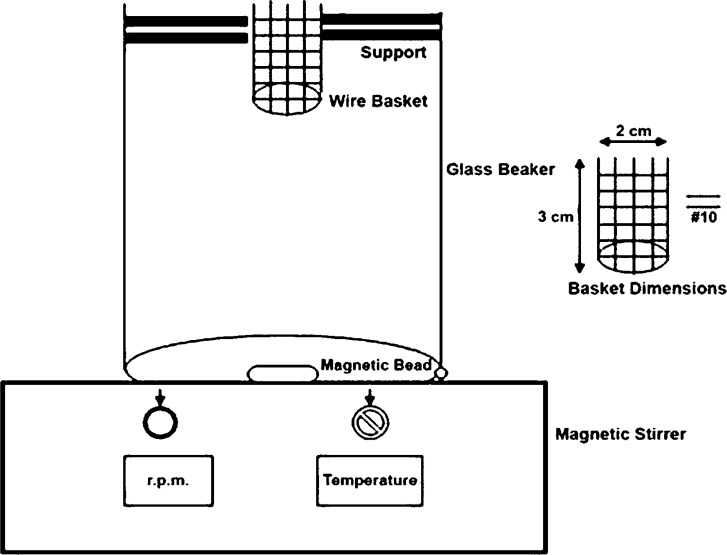
Modified disintegration test apparatus

*In-vitro *disintegration time for ODTs was determined using USP and modified disintegration test apparatus with pH 6.8 phosphate buffer as disintegrating medium (19). During this study we made an attempt to develop a more suitable apparatus for ODT because many reports indicated the unsuitability of conventional disintegration test apparatus for ODT (20, 21, 22, 23). Briefly, (Figure 1) the apparatus consisted of a glass beaker of 1000 mL capacity with the wire basket positioned in the beaker with the help of a support in a way that when the beaker contained 900 mL of phosphate buffer pH 6.8 as a disintegration medium, the basket had only 6 mL of it. A magnetic bead was placed at the bottom of a beaker and temperature was maintained at 37 ± 2 °C. Disintegration time was determined at 50 rpm (24). The results of two methods were compared.

*Weight variation*

20 tablets were selected at random and an average weight was determined (Electronic balance Adventure Ohaus, USA). Not more than two of individual weights deviated from the average weight by ± 7.5% (18).

*Uniformity of content*

Drug content from the tablets was determined by taking tablets from each formulation. Twenty tablets from each formulation were accurately weighed and powdered. Powder equivalent to 10 mg of drug was weighed and added into 100 mL volumetric flask. Then it was dissolved in 50 mL ethanol. The volume of solution was made to 100 mL (100 μg/mL). From this solution 1 mL was withdrawn and added into 10 mL volumetric flask and finally volume was made to 10 mL with phosphate buffer pH 6.8 (10 μg/mL), then solution was filtered with Whatman filter paper No. 41 and absorbance of the resulting solution was measured at 253.5 nm using UV spectrophotometer.

*Wetting time and water absorption ratio*

Wetting time is closely related to the inner structure of tablets and hydrophilicity of the excipients. According to the following equation proposed by washburn E.W (25), the water penetration rate into the powder bed is proportional to the pore radius and is affected by the hydrophilicity of the powders.

dl / dt = r γ cosθ / (4 ηl)

l = length of penetration 

r = capillary radius 

γ = surface tension 

η = liquid viscosity 

t = time 

θ = contact angle

A piece of a tissue paper folded twice was placed in a small petri plate (internal diameter 6.5 cm) containing 6 mL of water. A tablet was placed on the paper and time for complete wetting of the tablet was measured in seconds. The method was slightly modified by maintaining water at 37 ˚C. The same procedure was followed for determining the water absorption ratio. The wetted tablet was weighed and water absorption ratio, R, was determined according to the following equation:

R = { (Wa - Wb) / Wb } × 100 

Where, Wa and Wb were the weights of the tablet after and before study (24, 25).

*In-vitro dissolution study*

*In-vitro *dissolution of olanzapine ODTs was studied using Veego scientific, USP tablet dissolution apparatus type II. The dissolution was carried out in 900 mL phosphate buffer pH 6.8. at 37 ± 0.5 ˚C, at 50 rpm. 10 mL aliquots were withdrawn at specific time interval and filtered using Whatman filter paper No. 41. Absorbance of the filtered solution was checked UV spectrophotometrically at 253.5 nm and the drug content was determined. Sink conditions were maintained throughout the study. All studies were carried out in triplicate (3).

*Statistical analysis*

One way ANOVA with Students Paired ‘t’ test was applied to compare the dissolution rate of pure olanzapine with its inclusion complex and to compare the disintegration time between USP disintegration test apparatus and Modified disintegration test apparatus. One way ANOVA with Dunnett’s post test was applied to find out significant difference in time to release 100% olanzapine from different formulations.

## Results and Discussion

*Phase solubility analysis*

**Figure 2:**
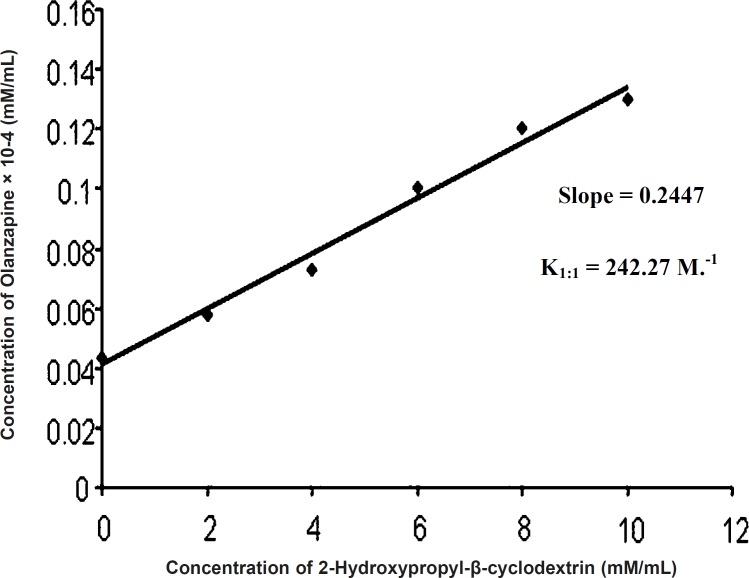
Phase solubility analysis of olanzapine-2-hydroxypropyl-β-cyclodextrin mixture

Phase solubility diagram can be classified as AL type (Figure 2) (5), as the solubility of the olanzapine almost linearly increased with increasing concentration of 2-hydroxypropyl-β-cyclodextrin (0 M-0.01 M). The increase in solubility in the system is due to molecular interactions between olanzapine and 2-hydroxypropyl-β-cyclodextrin to form complex. Because the straight line had a slope less than unity, it was assumed that increase in solubility was observed due to formation of 1:1 complex. The stability constant was found to be 242.27 M.^-1^ and value falling within the range of 50-2000 M.^-1^ , is to be adequate for the formation of inclusion complex, which may contribute to improve disssolution of poorly water soluble drugs (8). 

K_1:1 _= Slope/Intercept (1 - Slope) 

Slope = 0.2447

Intercept = 0.00134

K_1:1 _= 0.2447/0.00134 (1 - 0.2447) 

= 0.2447/0.00101 

= 242.27 M.^-1^

*Characterization of olanzapine-2-hydroxypropyl-β-cyclodextrin inclusion complex*

*Drug content estimation*

The drug content of inclusion complex prepared by kneading was found within the specification (98%-102%).

*Saturation solubility study*

In the present work, enhancement in the solubility was observed in case of inclusion complex. Solubility of pure olanzapine was found to be 13.13 ± 1.6 μg/mL and solubility of inclusion complex was found to be 22.97 ± 3.85 μg/mL.

*IR spectral analysis*

**Figure 3:**
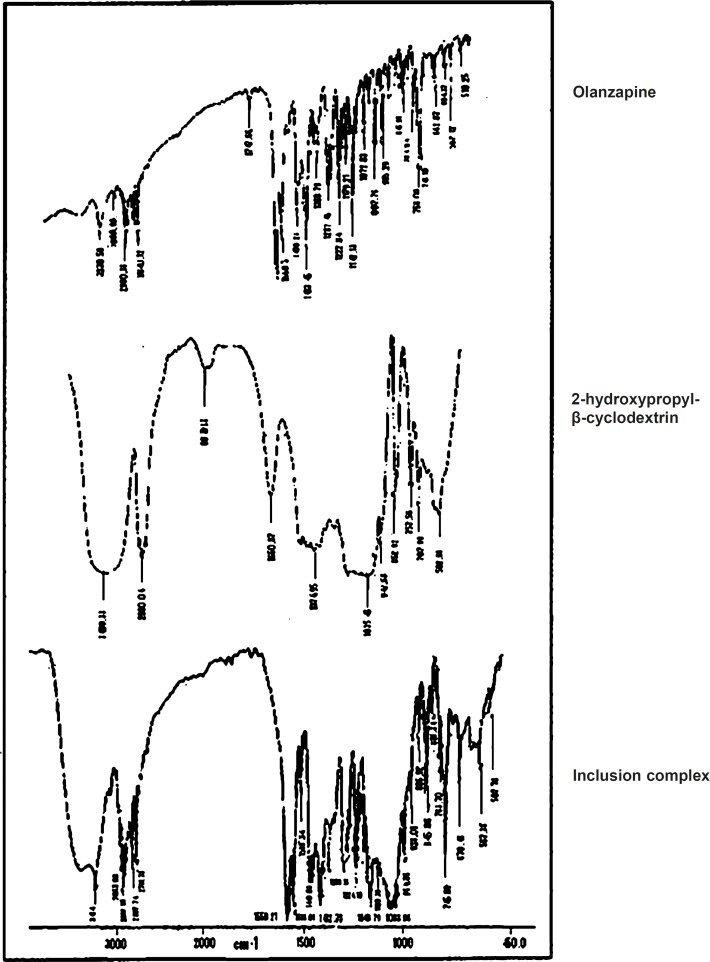
IR of olanzapine, 2-hydroxypropyl-β-cyclodextrin and inclusion complex

IR is a highly sensitive method of analysis, all spectra of complex showed some or other changes from parent spectra (Figure 3). Therefore complex formation can be assigned which could result in its inclusion into the hydrophobic cavity of 2-hydroxypropyl-β-cyclodextrin (13). It was observed that 1033.96 cm^-1^ due to O-H bending, 225.78 cm^-1^ due to N-H stretching, 2932.10 cm^-1^ due to C-H stretching, 1586.10 cm^-1^ due to C = C stretching, 1559.21 cm^-1^ due to C = N stretching, 745.88 cm^-1^ due to C-S bending. The IR spectrum of inclusion complex showed all the characteristic peaks of pure Olanzapine (26). 

*X-ray diffraction study*

**Figure 4:**
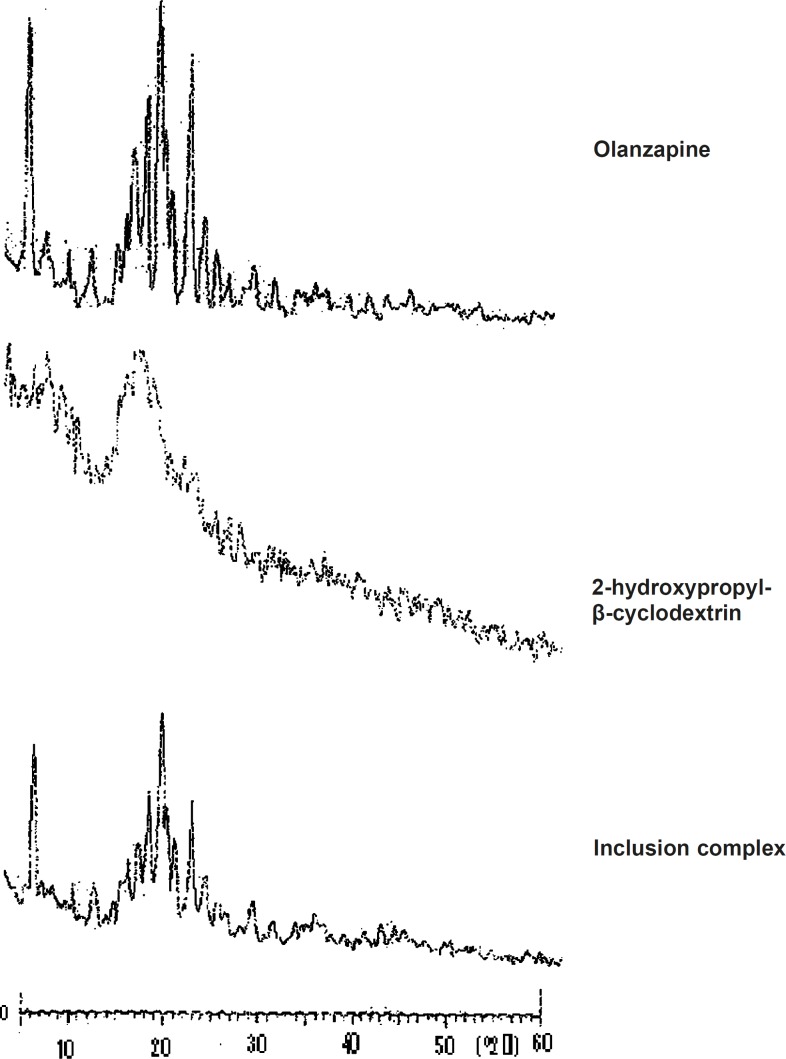
XRD of olanzapine, 2-hydroxypropyl-β-cyclodextrin and inclusion complex

X-ray diffraction patterns revealed the crystalline nature of pure olanzapine as well as the amorphous nature of 2-hydroxypropyl-β-cyclodextrin. The X-ray diffractogram of olanzapine (Figure 4) showed number of sharp and intense peaks. The diffractogram of 2-hydroxypropyl-β-- 

cyclodextrin showed diffused peaks indicating amorphous nature while the diffractogram of olanzapine-2-hydroxypropyl-β-cyclodextrin inclusion complex showed broad peaks with low intensity, suggesting probable transformation of micro crystalline form into an amorphous state (13). X-RD pattern of pure olanzapine showed principal peak at 20.87° and intense peaks at 8.48°, 18.22°, 19.69°, 21.39°, 23.84° and inclusion complex showed intense peaks at 20.95°, 8.58°, 19.85°, 21.45°, 22.21°, 23.89°. Increase in peak width was observed in X-RD pattern of inclusion complex.

*DSC-differential scanning calorimetry*

**Figure 5:**
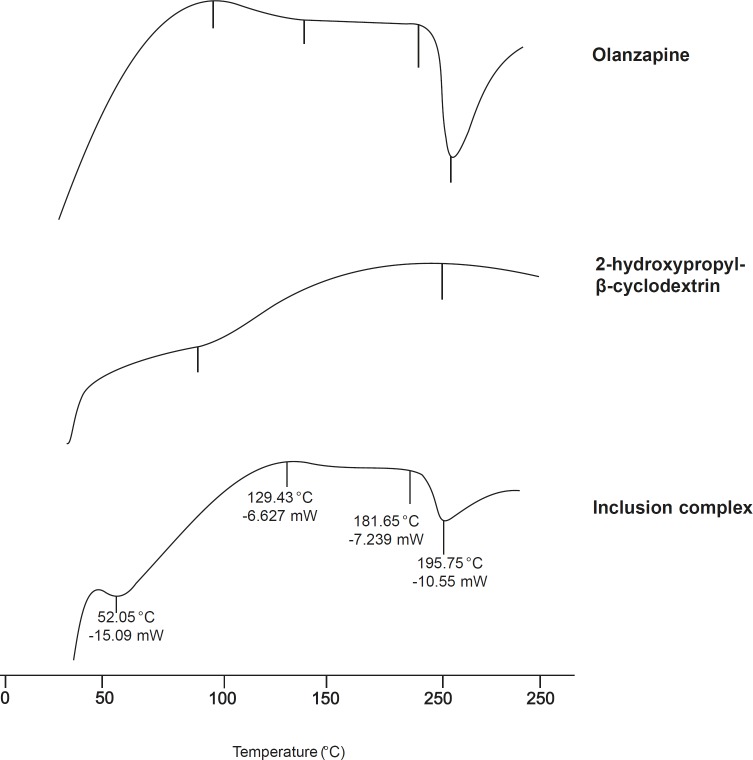
DSC of olanzapine, 2-hydroxypropyl-β-cyclodextrin and inclusion complex

Supporting evidence for complex formation was also obtained from DSC studies. The DSC thermograms (Figure 5), endothermic peak of olanzapine at 196.14 °C, which corresponds to its melting point, while 2-hydroxypropyl-β-cyclodextrin exhibited a typical broad endothermic peak at 87.12 °C. Broad endothermic peak at 50-100 °C assigned to its dehydration. The disappearance or shifting of endothermic or exothermic peaks of drugs is mostly an indication of formation of an inclusion complex (15, 27). The peak of 2-hydroxypropyl-β-cyclodextrin was shifted from 87.12-52.05 °C. In case of inclusion complex, intensity of the olanzapine melting endotherm had decreased. 

These observations clearly indicate strong evidences of inclusion of drug into cyclodextrin cavity.

*Dissolution study of olanzapine and its inclusion complex*

**Figure 6:**
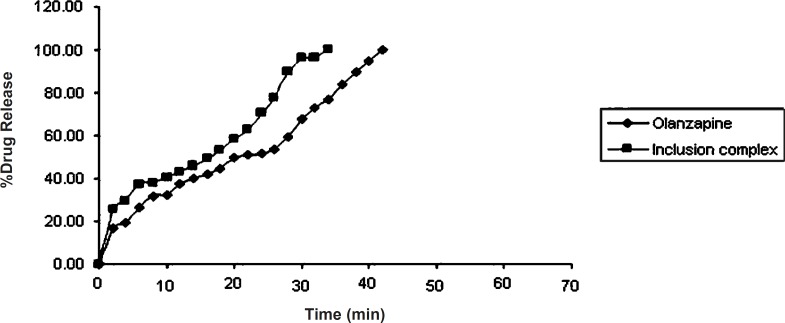
Drug release profiles of olanzapine and olanzapine-2-hydroxypropyl-β-cyclodextrin inclusion complex

From (Figure 6) it was observed that pure olanzapine had shown complete drug release (100.30 ± 0.37%) within 42 min while complex had shown complete drug release (99.70 ± 0.41%) within 34 min. Dissolution profile of olanzapine-2-hydroxypropyl-β-cyclodextrin was fast because of the hydroxypropyl-β-cyclodextrin complexation with drug. So dissolution rate of olanzapine was increased due to complexation with 2-hydroxypropyl-β-cyclodextrin. These findings coincide with the finding of Dahiya S, Pathak K, 2007 that cyclodextrin derivatives influence the release of aceclofenac from directly compressible tablets (28). 

*Evaluation of tablets*

The blends of all formulations showed good flowability and compressibility. From the results obtained (Table 2), it was observed that linear relationship exists between tablet crushing strength and hardness, as hardness goes on increasing tablet crushing strength also goes on increasing. Friability below 1% was an indication of good mechanical resistance of the tablets. From the hardness and friability study, it was reported that as the concentration of Avicel PH102 increases hardness goes on increasing and as the concentration of superdisintegrant increases friability goes on increasing. 

**Table 2:** Evaluation of tablets

**Formulations**	**Diameter** **(mm)**	**Thickness** **(mm)**	**Crushing strength** **(Kg/cm** ^2^ **)**	**Weight variation (%)**	**Hardness** **(kg/cm** ^2^ **)**	**Friability** **(%)**	**Uniformity of Content** **(%)**	**Wetting time** **(Sec.)**	**Water absorption Ratio (%)**	**Drug** **release (%)**
Control	7.90 ± 0.7	3.23 ± 0.42	0.094	1.34 ± 0.83	3.58 ± 0.21	0.47 ± 0.21	99.11 ± 0.23	57 ± 1.38	61 ± 1.42	99.15 ± 0.14 (30 Min.)
A1	7.93 ± 0.5	3.13 ± 0.74	0.090	1.98 ± 0.21	3.51 ± 0.32	0.78 ± 0.35	98.18 ± 0.72	13 ± 2.82	88 ± 3.87	98.15 ± 0.8 (20 Min.)
A2	7.50 ± 0.4	3.18 ± 0.34	0.087	1.44 ± 0.36	3.33 ± 0.38	0.82 ± 0.20	98.25 ± 1.10	12 ± 2.36	90 ± 1.36	98.35 ± 1.1 (16 Min.)
A3	7.82 ± 0.3	3.34 ± 0.8	0.085	1.57 ± 0.63	3.26 ± 0.21	0.87 ± 0.52	99.37 ± 0.52	9 ± 1.34	96 ± 1.28	99.72 ± 0.57 (6 Min.)
B1	7.82 ± 0.8	3.13 ± 0.92	0.092	1.51 ± 0.30	3.56 ± 0.28	0.85 ± 0.39	98.49 ± 0.29	12 ± 2.47	90 ± 2.52	98.61 ± 0.2 (16 Min.)
B2	7.94 ± 0.2	3.26 ± 0.64	0.088	1.46 ± 0.59	3.35 ± 0.13	0.85 ± 0.47	99.40 ± 0.46	11 ± 1.85	91 ± 1.47	99.85±0.3 (12 Min.)
B3	7.41 ± 0.31	3.21 ± 0.13	0.086	1.45 ± 0.81	3.24 ± 0.45	0.88 ± 0.10	99.20 ± 0.36	7 ± 1.12	104 ± 1.21	99.19 ± 0.18 (6 Min.)
C1	7.71 ± 0.12	3.14 ± 0.51	0.092	1.62 ± 0.23	3.55 ± 0.39	0.84 ± 0.31	98.59 ± 0.61	25 ± 1.34	78 ± 1.65	98.49 ± 0.6 (18 Min.)
C2	7.81 ± 0.18	3.14 ± 0.23	0.088	2.56 ± 0.31	3.42 ± 0.31	0.85 ± 0.27	99.12 ± 1.2	13 ± 1.53	86 ± 2.83	100.15 ± 0.21 (12Min.)
C3	7.84 ± 0.9	3.20 ± 0.59	0.086	2.64 ± 0.76	3.31 ± 0.58	0.86±0.25	99.24 ± 0.21	11 ± 1.88	91 ± 2.59	99.26 ± 0.26 (8 Min.)
D1	7.93 ± 0.6	3.76 ± 0.90	0.090	1.13 ± 0.20	3.50 ± 0.45	0.75 ± 0.40	99.27 ± 0.75	13 ± 3.21	86 ± 2.55	99.37 ± 0.83 (18 Min.)
D2	7.95 ± 0.2	3.64 ± 0.43	0.083	1.81 ± 0.48	3.18 ± 0.23	0.82 ± 0.24	98.83 ± 0.72	10±1.57	92 ± 2.19	98.99 ± 0.79 (14 Min.)
D3	7.93 ± 0.5	3.23 ± 0.32	0.090	1.51 ± 0.36	3.46 ± 0.17	0.79 ± 0.14	98.39 ± 0.49	10 ± 1.62	91 ± 1.35	98.43 ± 0.36 (16 Min.)
E1	7.89 ± 0.10	3.25 ± 0.50	0.089	1.13 ± 0.40	3.48 ± 0.38	0.52 ± 0.41	98.24 ± 0.34	14 ± 1.25	82 ± 3.72	98.35 ± 0.23 (20 Min.)
E2	7.74 ± 0.4	3.25 ± 0.35	0.086	1.94 ± 0.32	3.23 ± 0.82	0.75 ± 0.31	99.82 ± 0.17	12 ± 1.31	90 ± 2.38	99.95 ± 0.18 (16 Min.)
E3	7.81 ± 0.13	3.12 ± 0.54	0.089	1.94 ± 0.56	3.45 ± 0.45	0.67 ± 0.28	99.13 ± 0.5	29 ± 1.83	73 ± 2.77	100.15 ± 0.24 (22 Min.)

All values presented as mean ± SD (n = 3)

Exceptional case were of tulsion 339 and iIndion 414. The uniformity of content was found within acceptable limits. All the formulations passesd weight variation test. From disintegration test study (Table 3) it was found that significant difference was observed in disintegration time between USP disintegration test apparatus and Modified disintegration test apparatus. p value is 0.0004. In case of USP disintegration test apparatus, due to the increase in the volume of disintegration medium, tablet takes less time to disintegrate while in case of Modified disintegration test apparatus, due to decrease in the volume of disintegration medium, tablet takes more time to disintegrate. From the wetting time study it was reported that a linear relationship exists between wetting time and disintegration time. The wetting time is an important step for disintegration process to take place. By studying the water absorption ratio, it was reported that as the disintegration time decreases water absorption ratio increases.

**Table 3:** Comparative evaluation of disintegration time

**Formulations**	**D.T. measured by using USP D.T. apparatus (sec)**	**D.T. measured by using Modified D.T. apparatus (sec)**
Control	134 ± 2.9	290 ± 1.41
A1	27 ± 2.57	59 ± 3.21
A2	25 ± 1.62	54 ± 0.58
A3	20 ± 1.74	44 ± 2.51
B1	25 ± 2.62	59 ± 4.28
B2	25 ± 1.34	53 ± 1.23
B3	15 ± 1.27	39 ± 1.76
C1	55 ± 3.92	120 ± 4.67
C2	26 ± 2.75	55 ± 2.34
C3	21 ± 2.83	47 ± 3.45
D1	28 ± 1.80	64 ± 2.56
D2	21 ± 2.83	46 ± 3.76
D3	22 ± 3.12	49 ± 3.89
E1	31 ± 1.71	75 ± 3.45
E2	27 ± 1.47	57 ± 1.27
E3	68 ± 2.31	137 ± 1.54

All values presented as mean ± SD (n = 3)

**Figure 7:**
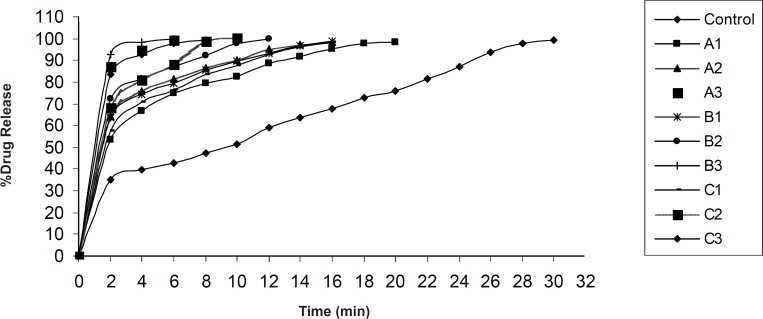
Drug release profiles of formulations control, A1, A2, A3 and B1, B2, B3, and C1, C2, C3 containing 8% wt/wt, 9% wt/wt and 10% wt/wt of sodium starch glycolate and 3% w/w, 4% w/w, and 5% wt/wt of croscarmellose sodium and crospovidone, respectively

**Figure 8:**
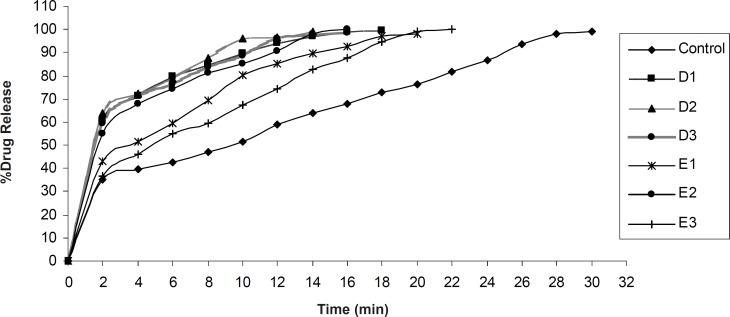
Drug release profiles of formulations control, E1, E2, E3 and D1, D2, D3 containing 3% wt/wt, 4% wt/wt and 5% wt/wt of tulsion 339 and indion 414, respectively

*In-vitro *drug release was found to be in the range of 98.15 ± 0.83% - 100.15 ± 0.24% (Figure 7, Figure 8). In each formulation it was observed that there was fast drug release at initial state of dissolution while in case of control formulation there was no initial rise in the dissolution rate. 

Significant difference was observed in dissolution rate of pure olanzapine with its inclusion complex, p < 0.0001 (Figure 6) because 2-hydroxypropyl-β-cyclodextrin forms the complex with olanzapine and increases it’s solubility and dissolution rate (28). The initial rise in the drug release was dependent upon the effectivity and concentration of superdisintegrant. The bursting effect of superdisintegrant showed rise (shoot) in the drug release. From this study it was reported that decrease in the disintegration time showed faster drug release. In case of formulation D1, D2, D3 and E1, E2, E3, by increasing the concentration of tulsion 339 and indion 414, disintegration time increases. This was due to concentration of superdisintegrant crossing the critical concentration of superdisintegrant, so more amount of phosphate buffer required to swell and to disintegrate the tablet. Also it was reported that tulsion 339 and indion 414 showed poor results of disintegration time because these superdisintegrants have highly crosslinked structure as compared to other superdisntegrants. So these superdisintegrants showed high disintegration time. Resin blocks the pores of tablets, so it takes more time to disintegrate (10). Formulation A2 and A3 showed superior disintegration time of less than one min, but tablets did not disintegrate with formation of granules which is desired for ODTs (29, 30). Formation of lumps were observed during disintegration of the tablets. Formulation A1 showed no formation of lumps and tablets were disintegrated in a granular way. Formulation A3 showed superior result of disintegration time and showed faster drug release as compared to formulation A1 and A2. Formulation B3 showed superior result of disintegration time and showed faster drug release as compared to formulation B1 and B2. Formulation C3 showed superior result of disintegration time and faster drug release as compared to formulation C1 and C2. Formulation D2 showed superior result of disintegration time and showed faster drug release as compared to formulation D1 and D3. Formulation E2 showed superior result of disintegration time and showed faster drug release as compared to formulation E1 and E3.

Control formulation showed 290 ± 1.41 sec disintegration time and 99.15 ± 0.14% drug release. Among all the formulations, 5% wt/wt croscarmellose sodium has given excellent results. These findings are supported by the studies reported by Amrutkar JR *et al*. 2007. Authors have reported that tablets prepared with croscarmellose sodium proved best among all superdisintegrants (10). These findings also coincide with the conclusion of Shirwaikar AA *et al. *2004 that croscarmellose sodium showed faster disintegration and subsequent drug release (31).

Significant difference was found in time to release 100% olanzapine from different formulations, p < 0.0001. Dunnett’s comparison test showed all formulations are superior in drug release as compared to control formulation. B3 formulation containing croscarmellose sodium as superdisintegrant was superior as compared to other formulations. Mean difference control vs. B3 was 32.20. It is made by crosslinking (etherification) reaction of sodium CMC. This crosslinking greately reduces the water solubility of sodium CMC while permeates material to swell and absorbs water many times to its weight without loosing its fiber integrity. It swells to large extent to disintegrate the tablets and has fibrous nature that allows interparticulate as well as extraparticulate wicking of water (29, 30). It was observed that as the concentration of croscarmellose sodium increases water absorption ratio increases and *in-vitro *disintegration time decreases. Formulation B3 showed 39.40 ± 1.76 sec disintegration time and 99.19 ± 0.18% drug rerelease within 6 min.

From the observations, it was evident that water uptake capacity of superdisintegrant follows in the order of croscarmellose sodium > SSG > crospovidone > tulsion 339 > indion 414. All the formulations showed superior results as compared to control formulation.

## Conclusion

Phase solubility analysis data suggests 1:1 complex formation of olanzapine with 2-hydroxypropyl-β-cyclodextrin. Inclusion complex showed increase in saturated solubility and dissolution rate of the drug. Type of superdisintegrant and concentration of superdisintegrant affect the drug release and disintegration time. Combination of 2-hydroxypropyl-β-cyclodextrin and super-disintegrant in formulations showed excellent results. It gave the faster drug release and enhancement in the dissolution rate. Croscarmellose sodium gave better disintegration characteristics than other superdisintegrants. However, further studies like stability studies and *in-vivo *studies are needed to develop the formulation. 
